# Deletion of NAD(P)H Oxidase 2 Prevents Angiotensin II-Induced Skeletal Muscle Atrophy

**DOI:** 10.1155/2018/3194917

**Published:** 2018-01-02

**Authors:** Tomoyasu Kadoguchi, Shingo Takada, Takashi Yokota, Takaaki Furihata, Junichi Matsumoto, Masaya Tsuda, Wataru Mizushima, Arata Fukushima, Koichi Okita, Shintaro Kinugawa

**Affiliations:** ^1^Department of Cardiovascular Medicine, Faculty of Medicine and Graduate School of Medicine, Hokkaido University, Kita-15, Nishi-7, Kita-ku, Sapporo, Hokkaido 060-8638, Japan; ^2^Graduate School of Lifelong Sport, Hokusho University, 23 Bunkyodai, Ebetsu, Hokkaido 069-8511, Japan

## Abstract

Skeletal muscle atrophy is induced by an imbalance between protein synthesis and degradation. Our previous studies reported that angiotensin II (AII) directly induced muscle atrophy in mice. This study investigated the role of NAD(P)H oxidase 2 (Nox2) activation by AII in the induction of skeletal muscle atrophy. For 4 weeks, either saline (vehicle: V) or AII (1000 ng kg^−1^ min^−1^) was infused into male wild-type (WT) and Nox2 knockout (KO) mice via osmotic minipumps. Experiments were performed in the following 4 groups: WT + V, KO + V, WT + AII, and KO + AII. Body weight, muscle weight, and myocyte cross-sectional area were significantly decreased in WT + AII compared to WT + V mice, and these changes were not observed in KO + AII mice. Akt phosphorylation of Ser473 and p70S6K of Thr389 was decreased, gene expression levels of MuRF-1 and atrogin-1 were increased in WT + AII compared to WT + V, and these changes were significantly attenuated in KO + AII mice. The deletion of Nox2 prevented AII-induced skeletal muscle atrophy via improving the balance between protein synthesis and degradation. Therefore, Nox2 may be a therapeutic target for AII-induced skeletal muscle atrophy.

## 1. Introduction

The pathogenesis of sarcopenia, which is characterized by the loss of muscle mass and strength and low physical performance, has been recently attracting attention [1]. The occurrence of sarcopenia is linked to aging, although other conditions such as physical inactivity, poor nutrition, endocrine abnormalities, and heart failure (HF) are also involved in its pathogenesis. Sarcopenia directly lowers physical activity and adversely affects disease prognosis; thus, investigating the mechanisms associated with a decrease in muscle mass is of great importance.

Skeletal muscle atrophy, a primary component of sarcopenia, can be caused by physical inactivity, catabolic steroids such as glucocorticoids, inflammatory cytokines, reactive oxygen species (ROS), and catabolic nutritional states [2–4]. It is known that either skeletal muscle atrophy or hypertrophy may occur as a result of an imbalance between protein synthesis and degradation [5]. Protein degradation is regulated by several catabolic transcription factors such as the forkhead box (FoxO) and nuclear factor-kappa B (NF-*κ*B) proteins. FoxO and NF-*κ*B regulate the transcription of atrogenes and E3 ubiquitin ligases including muscle RING Finger-1 (MuRF-1) and MAFbx/atrogin-1 (atrogin-1). On the other hand, protein synthesis is mainly regulated by the phosphoinositide 3-kinase- (PI3K-) Akt cascades. This pathway also inhibits the degradation pathway mediated by FoxO protein and atrogenes [2].

It has been well documented that the excess activation of the renin-angiotensin system (RAS) plays a central role in aging and the pathogenesis and progression of various chronic diseases such as diabetes and HF [6–10]. Angiotensin II (AII), a main effector molecule of the RAS, plays an important role in these processes. Although the mechanisms of these conditions are complex and not well studied, muscle wasting syndromes such as sarcopenia are associated with elevated AII [11]. We have previously demonstrated that AII can directly induce skeletal muscle atrophy via a decrease in Akt phosphorylation, a key molecule of protein synthesis, as well as an increase in MuRF-1 and atrogin-1 expression, key molecules of protein degradation [12]. Additionally, AII activates NAD(P)H oxidase (Nox) and increases Nox-derived ROS via the AII type I receptor [13]. Previous studies reported that gene expression of Nox2 was increased in the skeletal muscle from mice with myocardial infarction [14–16]. Nox2 is the main isoform of NADPH oxidase responsible for superoxide anion generation [17]. Therefore, we hypothesized that AII-induced Nox activation was associated with AII-induced skeletal muscle atrophy. The purpose of this study was to investigate the effect of Nox2 deletion on the prevention of AII-induced skeletal muscle atrophy.

## 2. Materials and Methods

### 2.1. Animal Model

Male C57BL/6J and Nox2-deficient (KO) (B6.129S-Cybb^tm1Din^/J, Stock number 002365, gp91^phox^-, Jackson Laboratory, Bar Harbor, Maine) mice (8–12 weeks of age) were provided with chow and water ad libitum and housed in pairs on a fixed 12-h light/dark cycle. At baseline, there were no differences in physical characteristics between WT and Nox2 KO mice. An osmotic minipump (Alzet model 2004, Alza Corporation, Palo Alto, Calif) was implanted under tribromoethanol/amylene hydrate (Avertin; 2.5% wt/vol, 10 *μ*L/g body weight, i.p.) anesthesia to infuse AII (1000 ng kg^−1^ min^−1^) continuously for 4 weeks. Saline was used as vehicle. Mice were randomly divided into following 4 groups; WT + V (*n* = 8), KO + V (*n* = 6), WT + AII (*n* = 8), and KO + AII (*n* = 6). All experiments were performed under barrier condition. These assignment procedures were performed using numeric codes to identify the animals. All procedures and animal care were approved by our institutional animal research committee and conformed to the animal care guidelines for the Care and Use of Laboratory Animals at Hokkaido University Graduate School of Medicine.

### 2.2. Blood Pressure Measurement and Organ Weight

Systemic blood pressure was measured by using tail-cuff method (BP-98A; Softron, Tokyo, Japan) without anesthesia. Mice were killed by cervical dislocation under deep anesthesia with Avertin. Heart and unilateral hindlimb skeletal muscle were then excised and weighted. Total hindlimb skeletal muscle was used in all experiments.

### 2.3. Histology in the Skeletal Muscle

Hindlimb skeletal muscle was excised, fixed in 4% paraformaldehyde, embedded in paraffin, and stained with hematoxylin-eosin (HE) for histological analysis. Morphological analysis of muscle cross-sectional area was performed in at least 50 cells from each mouse [12, 18, 19].

### 2.4. Immunoblotting in the Skeletal Muscle

Immunoblotting was performed as previously described [8–10, 12, 18–23]. Briefly, gastrocnemius muscle tissue samples were homogenized in 1x cell lysis buffer (Cell Signaling, Danvers, MA), supplemented with 1x complete protease inhibitor cocktail (Roche, Basel, Switzerland) and 1 mmol/l phenyl methyl sulphonic fluoride. After sonification and centrifugation at 15,000*g* for 10 min at 4°C, the supernatants were collected. Protein aliquots were taken for total protein assay (Pierce BCA, Rockford, IL), and the remaining lysate of 20 *μ*g was added onto 4–20% gradient or AnykD gels (Bio-Rad, Hercules, CA), electrophoretically separated by sodium dodecyl sulfate-polyacrylamide gel using running buffer, and transferred by electroblotting to a polyvinylidene fluoride membrane (Bio-Rad) using transfer buffer at 80 V for 2 hours. After the membranes were blocked in Tris-buffered saline buffer with 0.1% Tween-20 (TBST) in 5% nonfat dry milk, they were incubated overnight at 4°C with primary antibodies (dilution 1 : 1,000) against the phosphorylated forms of phosphoserine Akt (p-Akt) and phosphothreonine p70 ribosomal S6 kinase (p-p70S6K), Akt, cleaved caspase-3 (Cell signaling), MuRF-1, atrogin-1 and p70S6K (Santa Cruz Biotechnology, Santa Cruz, CA). Equal loading of protein was verified by immunoblotting with glyceraldehyde-3-phosphate dehydrogenase (GAPDH) (Cell Signaling). After being washed three times in TBST buffer, the membranes were incubated with secondary antibodies conjugated with horseradish peroxidase (dilution 1 : 5,000; Santa Cruz, Santa Cruz Biotechnology, CA). The membranes were washed again in TBST and incubated with the chemiluminescence detection reagent in the Amersham ECL Western Blotting Analysis System (GE Healthcare, Chalfont St Giles, United Kingdom) for enhanced chemiluminescence. Proteins were quantified (band × volume) using Molecular Imager® ChemiDocTM XRS Plus system in combination with Image LabTM Software (ver. 2.0; Bio-Rad).

### 2.5. O_2_^−^ Production and NAD(P)H Oxidase Activity

The chemiluminescence elicited by O_2_^−^ in the presence of lucigenin (5 *μ*mol/l) was measured in gastrocnemius muscle using a luminometer (AccuFLEX Lumi 400; Aloka, Tokyo, Japan) as previously described with some modifications [8–10, 22, 24–26]. To validate that the chemiluminescence signals were derived from O_2_^−^, the measurements were also performed in the presence of tiron (20 mmol/l), a cell-permeant, nonenzymatic scavenger of O_2_^−^. NAD(P)H oxidase activity was measured in the homogenates isolated from gastrocnemius muscle by the lucigenin assay after the addition of NAD(P)H (300 *μ*mol/l) as previously described [8–10, 21, 22, 24, 25].

### 2.6. Apoptosis

To detect apoptosis, skeletal muscle tissue sections were stained with terminal deoxynucleotidyl transferase-mediated dUTP nick end labeling (TUNEL). The number of TUNEL-positive skeletal muscle myocyte nuclei was counted, and the data were normalized per 100 total nuclei identified by hematoxylin-positive staining in the same sections as described previously [12].

### 2.7. Exercise Tolerability

Exercise tolerability with treadmill was performed to measure indices defining whole body exercise capacity as previously described [8, 21–24]. At the time of treadmill testing, each mouse was placed on a treadmill (Oxymax 2; Columbus Instruments, Columbus, OH). After acclimation period for 10 min, mice were then provided with a 10 min warm-up period at 6 m/min at zero degrees. After the animals warmed up, the angle was fixed at 10 degrees and the speed was incrementally increased by 2 m/min every 2 min until the mouse reached exhaustion. Exhaustion was defined as spending time (10 sec) on the shocker plate without attempting to reengage the treadmill. This treadmill protocol was designed to linearly increase oxygen uptake in mice, which would attain plateau at the time of exhaustion, on the basis of previous our and other studies. Work was defined as the product of the vertical running distance to exhaustion and body weight.

### 2.8. Citrate Synthase and Mitochondrial Complex Activities in the Skeletal Muscle

Activity of citrate synthase (CS; a key enzyme of tricarboxylic acid cycle) was spectrophotometrically determined in the tissue homogenate from skeletal muscle sample, as described previously. The specific enzymatic activities of electron transport chain (ETC) complex I (rotenone-sensitive NADH-ubiquinone oxidoreductase) and complex III (ubiquinol-cytochrome-c oxidoreductase) were also measured in the mitochondria isolated from skeletal muscle, as described previously [21–24].

### 2.9. Statistical Analysis

Results are expressed as means ± SEM. For multiple-comparisons, two-way ANOVA followed by Tukey's test was performed. A value of *P* < 0.05 was considered statistical significant.

## 3. Results

### 3.1. Animal Characteristics

Table 1 showed animal characteristics in all groups after 4 weeks. Heart weight, heart weight/body weight, systolic blood pressure, diastolic blood pressure, and mean blood pressure were significantly higher in WT + AII than WT + V (Table 1), indicating that AII induced hypertension and cardiac hypertrophy. There were no significant differences in these parameters between WT + AII and KO + AII. There was no significant difference in heart rate among all groups (Table 1).

### 3.2. Skeletal Muscle Atrophy

**Figure 1:**
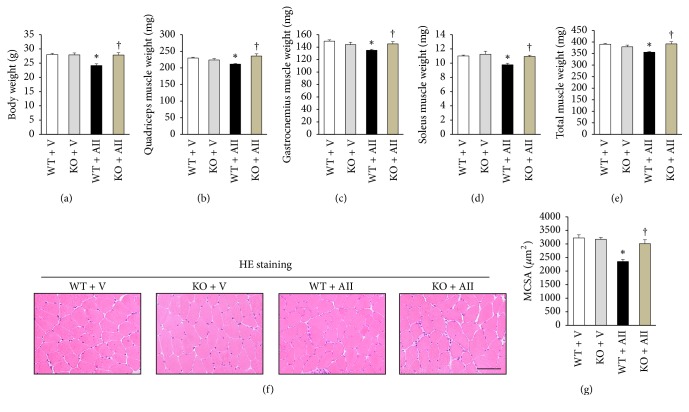
*Deletion of Nox2 prevents angiotensin II- (AII-) induced skeletal muscle atrophy*. (a) Body and lower limbs skeletal muscle weights (Quadriceps (b), gastrocnemius (c), soleus (d), and total muscle (e)) from WT + V, KO + V, WT + AII, and KO + AII mice after 4 weeks (*n* = 6–8 for each group). Representative high-power photomicrographs of skeletal muscle tissue sections stained with hematoxylin-eosin (HE) from 4 groups of mice (f) and summary data of myocyte cross-sectional area (g) (*n* = 5 for each group). Scale bar, 100 *μ*m. Data are expressed as means ± SEM. ^*∗*^ *P* < 0.05 versus WT + V. ^†^ *P* < 0.05 versus WT + AII. WT, wild type; V, vehicle; KO, knockout.

Body and lower limb skeletal muscle weights (quadriceps, gastrocnemius, soleus, and total) were significantly lower in WT + AII than WT + V, and these changes were attenuated in KO + AII (Figures 1(a)–1(e)).  Figure 1(f) showed representative images of muscle stained with HE. The myocyte cross-sectional area was significantly smaller in WT + AII than WT + V, and it was restored in KO + AII compared to WT + AII (Figures 1(f) and 1(g)). On the other hand, there were no differences in skeletal muscle weight adjusted with body weight (data not shown).

### 3.3. Protein Synthesis and Degradation Markers in the Skeletal Muscle

**Figure 2:**
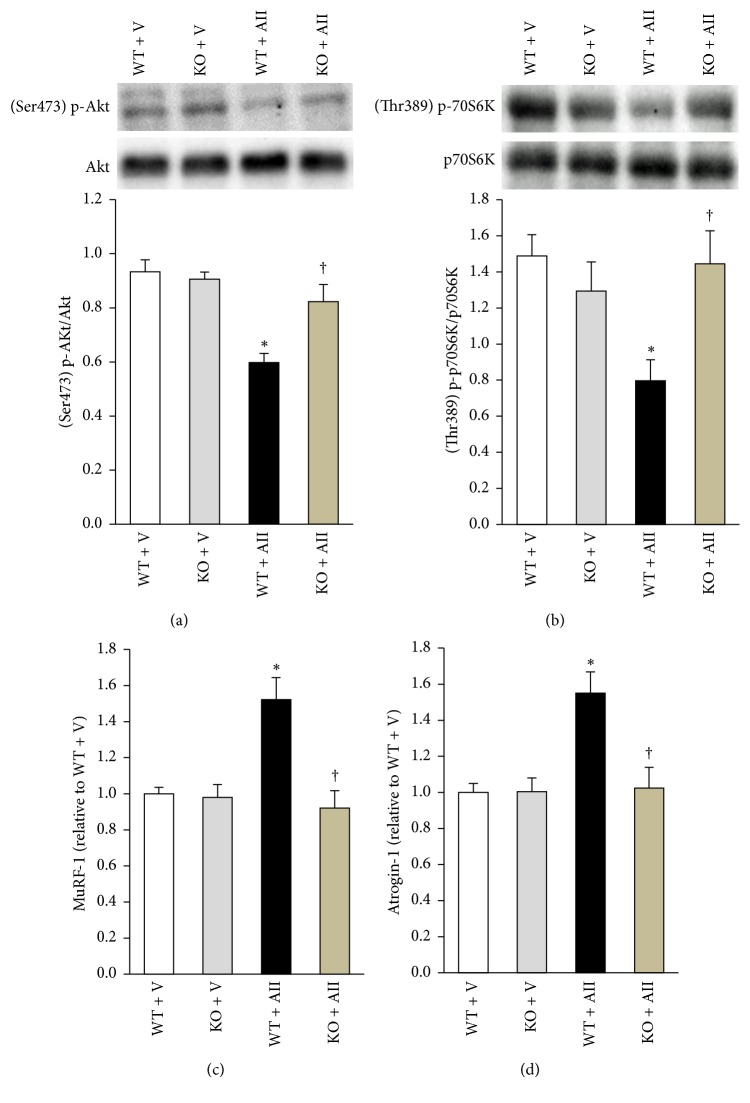
*Deletion of Nox2 regulates the balance between protein synthesis and degradation*. Representative Western blot and the summary data of quantitative analysis of protein expressions of p-Akt (Ser473 (a)) and p-p70S6K (Thr389 (b)) and summary data of gene expressions of MuRF-1 (c) and atrogin-1 (d) in the skeletal muscle tissue obtained from WT + V, KO + V, WT + AII, and KO + AII mice (*n* = 6 for each group). Data are expressed as means ± SEM. ^*∗*^ *P* < 0.05 versus WT + V. ^†^ *P* < 0.05 versus WT + AII. p-p70S6K, phosphorylation of p70 ribosomal S6 kinase; MuRF-1, Muscle RING Finger-1; atrogin-1, muscle atrophy F-box.

Representative images of western blot analysis for protein synthesis markers were shown in Figures 2(a) and 2(b). Akt phosphorylation of Ser473 and p70S6K of Thr389 were decreased in WT + AII compared to WT + V, and these changes were significantly attenuated in KO + AII (Figures 2(a) and 2(b)). mRNA expression levels of MuRF-1 and atrogin-1, key molecules of protein degradation, were significantly increased in WT + AII compared to WT + V, and they were also restored in KO + AII (Figures 2(c) and 2(d)).

### 3.4. O_2_^−^ Production and NAD(P)H Oxidase Activity in the Skeletal Muscle

**Figure 3:**
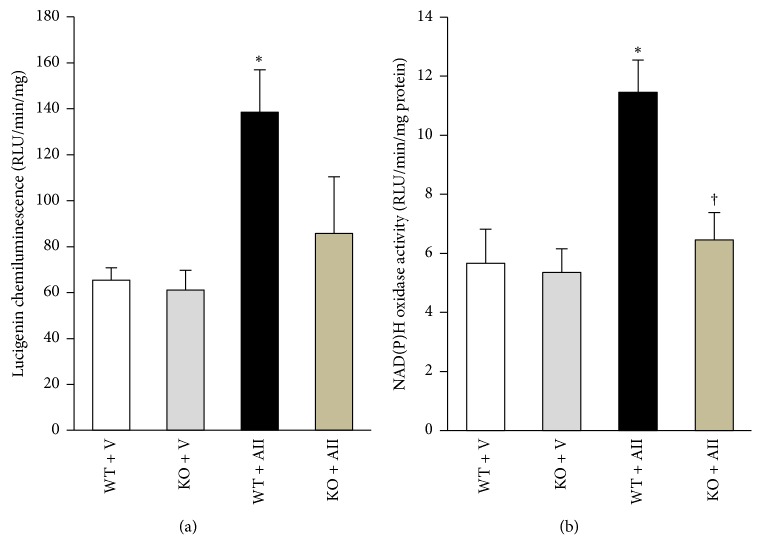
*Angiotensin II-Induced increase in NAD(P)H oxidase activity and superoxide production was prevented by Nox2 Knockout*. Superoxide production (*n* = 5–7 for each group (a)) and NAD(P)H oxidase activity (*n* = 5–7 for each group (b)) in the skeletal muscle tissue from WT + V, KO + V, WT + AII, and KO + II mice (*n* = 6 for each group). Data are expressed as means ± SEM. ^*∗*^ *P* < 0.05 versus WT + V. ^†^ *P* < 0.05 versus WT + AII.

O_2_^−^ production and NAD(P)H oxidase activity measured by lucigenin chemiluminescence in the skeletal muscle were significantly increased in WT + AII compared to WT + V (Figure 3). Although O_2_^−^ production tended to restore (Figure 3(a)), NAD(P)H oxidase activity was completely decreased in KO + AII (Figure 3(b)).

### 3.5. Apoptosis in the Skeletal Muscle

**Figure 4:**
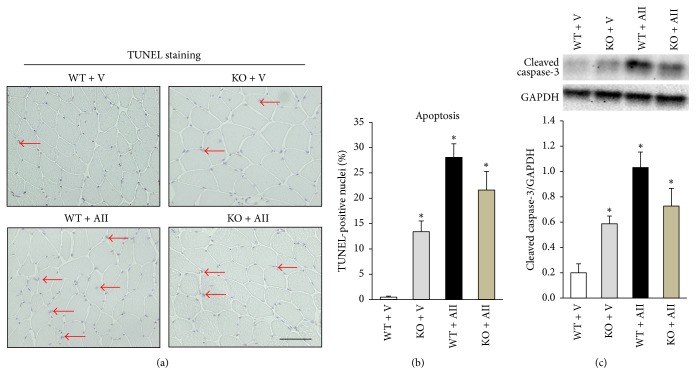
*AII-induced apoptosis in the skeletal muscle is not improved by Nox2 deletion*. Representative high-power photomicrographs of skeletal muscle tissue sections stained with terminal deoxynucleotidyl transferase-mediated dUTP nick end labeling (TUNEL) and summary data of quantitative analysis of TUNEL-positive nuclei ((a), (b) *n* = 5 for each group). Scale bar, 100 *μ*m. Quantitative analysis of cleaved (activated) caspase-3 in skeletal muscle tissue from WT + V, KO + V, WT + AII, and KO + AII ((c) *n* = 6 for each group). Data are expressed as means ± SEM. ^*∗*^ *P* < 0.05 versus WT + V.

Figure 4(a) showed representative images of staining for TUNEL. TUNEL-positive nuclei in the skeletal muscle were significantly increased in KO + V, WT + AII, and KO + AII compared to WT + V and there was no significant difference between WT + AII and KO + AII (Figure 4(b)). Similarly, the protein expression level of cleaved caspase-3 was significantly increased in KO + V, WT + AII, and KO + AII compared to WT + V and there was no significant difference between WT + AII and KO + AII (Figure 4(c)).

### 3.6. Exercise Tolerability

**Figure 5:**
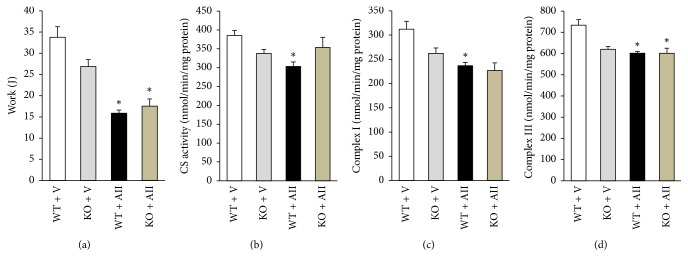
*Ang II-induced reduction of exercise tolerability is not improved and mitochondrial dysfunction in the skeletal muscle is not inhibited by Nox2 deletion*. (a) Work (*n* = 7-8 for each group), (b) citrate synthase activity, (c) complex I activity, and (d) complex III activity (*n* = 6 for each groups) in the isolated mitochondria from the skeletal muscle from WT + V, KO + V, WT + AII, and KO + AII mice. Data are expressed means ± SE. ^*∗*^ *P* < 0.05 versus WT + V.

Figure 5(a) showed whole body exercise tolerability measured by treadmill test. The work, run times, and run distance to exhaustion were significantly decreased in WT + AII compared to WT + V mice, and there were no significant changes in these parameters between WT + AII and KO + AII (Figure 5(a)).

### 3.7. Mitochondrial Function in the Skeletal Muscle

CS activity was significantly decreased in WT + AII compared to WT + V. Mitochondrial ETC complex I and III activities in the isolated mitochondria from skeletal muscle were also significantly decreased in WT + AII compared to WT + V (Figures 5(b)–5(d)). There were no significant differences in CS activity and complex activities between WT + AII and KO + AII (Figures 5(b)–5(d)).

## 4. Discussion

This is the first study to reveal that the deletion of Nox2 prevented AII-induced skeletal muscle atrophy. This was accomplished by restoring the protein synthesis and degradation. Our data indicated that Nox2-derived O_2_^−^ production played a central role in AII-induced skeletal muscle atrophy. Therefore, Nox2-dependent ROS production may be a therapeutic target for AII-induced skeletal muscle atrophy.

Our previous study demonstrated that a pressor dose of AII (1000 ng kg^−1^ min^−1^) continuously infused into mice for 4 weeks induced skeletal muscle atrophy [12]. Moreover, increases in ROS production and Nox activation were observed in the skeletal muscle of the same animal model [12]. However, the cause-and-effect relationship between AII-induced skeletal muscle atrophy and Nox-derived ROS has been undetermined. In the present study, it was clearly demonstrated that the deletion of Nox2 almost completely inhibited AII-induced skeletal muscle atrophy with weight loss (Figures 1(a)–1(e)). On the other hand, we could not evaluate muscle strength. Kackstein et al. observed that treatment of AII in mice decreased muscle strength and concomitantly increased Nox2-derived ROS production [27]. This suggests that AII-induced decrease in muscle strength is also inhibited by Nox2 deletion. Increased blood pressure and heart weight/body weight by AII were not inhibited in Nox2 KO mice (Table 1). These results indicated that the development of AII-induced skeletal muscle atrophy was independent of systemic blood pressure and cardiac hypertrophy. In our previous study, AII-induced skeletal muscle atrophy was the result of both a decrease in protein synthesis and an increase in protein degradation in the skeletal muscle [12].

Bodine et al. reported that deletion of MuRF1 or atrogin-1 suppressed muscle atrophy by denervation via inhibiting protein degradation [28]. Cho et al. reported that deletion of Akt or S6K1 induced muscle atrophy via suppressing protein synthesis [29, 30]. Therefore, these are reasonable markers that evaluate imbalance of protein in the skeletal muscle. Indeed, these markers have been used in many studies [31–34]. In the present study, the decreases in Akt and p70S6K phosphorylation and the increases in MuRF-1 and atrogin-1 expression were restored in Nox2 KO mice (Figure 2). It is well known that AII leads to Nox2-derived ROS production in various cells including the skeletal muscles [13, 35]. These results indicated that Nox2-derived ROS production caused AII-induced skeletal muscle atrophy through the disruption of protein synthesis and the induction of protein degradation in this model.

The present data demonstrated that ROS production reduced Akt phosphorylation. Studies previously demonstrated that the activation of Nox2 also decreased Akt phosphorylation at Ser473 in the skeletal myocytes such as C2C12 and L6 myotubes [36]. Several signaling pathways are considered to be involved in the decreased Akt phosphorylation. Myostatin is a transforming growth factor-*β* family member acting as a negative regulator of skeletal muscle growth. It is demonstrated that myostatin signals negatively regulate Akt/mammalian target of rapamycin (mTOR)/p70S6K signaling through activin receptor II/activin receptor-like kinase receptor complex, leading to the repression of protein synthesis [37, 38]. AII is known to enhance myostatin expression in cultured rat neonatal cardiomyocytes [39]. On the other hand, it has never been known whether ROS induce myostatin expression in the skeletal muscle. Wei et al. reported that this was due to a decreased insulin receptor substrate 1 (IRS-1) phosphorylation. The most important mechanism that leads to protein synthesis is a signal mediated by Insulin-like growth factor- (IGF-) 1. It positively regulates Akt/mTOR/p70S6K signaling through IGF-1 tyrosine kinase receptor and IRS-1 phosphorylation. It has been reported that ROS can induce a decrease in IGF-1 gene expression in cultured cells [40]. Our preliminary data showed that there were no differences in myostatin, IGF-1, and IRS-1 gene expression levels in skeletal muscle between vehicle and AII mice (data not shown). Hence, Akt phosphorylation may be impaired by a direct effect of Nox-derived ROS or other signaling pathways.

In the present study, the increase in MuRF-1 and atrogin-1 expression was restored. It is well known that protein degradation is involved in AII-induced skeletal muscle atrophy. Previous report suggests that protein degradation may be prevented by the antioxidants butylated hydroxytoluene and diphenyleneiodonium in myotubes [41]. Furthermore, it has been demonstrated that AII-induced muscle atrophy and 20S proteasome activity are attenuated in p47^phox^ deficient mice [42]. The regulation of E3 ubiquitin ligases, MuRF-1, and atrogin-1 by ROS production may be associated with these processes. Indeed, it has been reported that exposure to hydrogen peroxide can induce an increase in MuRF-1 and atrogin-1 expression in myotubes [43]. Additionally, skeletal muscle atrophy and proteasome activity developed in a mouse model of myocardial infarction were prevented by the Nox inhibitor, apocynin [15]. These were accompanied by the inhibition of NF-*κ*B and p38MAPK, both of which are known to be inducers of E3 ubiquitin ligases activation [44, 45]. Furthermore, inflammatory cytokines, especially tumor necrosis factor-*α* (TNF-*α*), play an important role in protein degradation. In fact, numerous atrophy models show an increase in this inflammatory cytokine. TNF-*α* activates NF-kB and increases MuRF-1 gene expression [46]. The interaction between ROS and inflammatory cytokines is well known, and the exposure of cultured cells to hydrogen peroxide can also increase TNF-*α* [47]. However, our preliminary data showed that IL-6 and TNF-*α* gene expression levels in the skeletal muscle were not altered after AII infusion (data not shown). These results indicate that Nox-derived ROS but not inflammatory cytokines are of great importance to protein degradation and AII-induced skeletal muscle atrophy.

Our previous study revealed that apoptotic cell death and cleaved caspase-3 significantly increased in a time-dependent manner during AII infusion, which might be associated with mitochondrial dysfunction [12]. In the present study, apoptotic cell death and cleaved caspase-3 were not restored in Nox2 KO mice. Moreover, Nox2 deletion did not restore AII-induced mitochondrial dysfunction and limited endurance exercise tolerability (Figure 5). In general, excess ROS production has been closely associated with the induction of apoptotic cell death. However, our data suggest that apoptotic cell death and mitochondrial dysfunction are independent of Nox2-induced ROS. On the other hand, we cannot clearly reveal the reason for increases in apoptotic cell death and caspase-3 activation in Nox2-deficient mice. We speculate that the elimination of physiological ROS production by deletion of Nox2 might lead to apoptotic cell death in the skeletal muscle. However, significant apoptotic cell death could be compensated by active regeneration ability, which is characteristic of skeletal muscle.

Finally, the Nox2 deficient mice have been used as a model of chronic granulomatous disease [48]. Indeed, we previously reported that the development of abscesses and/or invasion of inflammatory cells occurred in lungs as well as livers from these mice; in contrast, no inflammation was observed in heart and kidney [49]. In the present study, no histological evidence of chronic inflammation was not shown in skeletal muscle (Figure 1(f)). Therefore, the role of Nox2 is different among organs and inflammation of other organs does not influence the results of our study.

## 5. Conclusions

Skeletal muscle atrophy is a major contributor to negative outcomes in conditions such as aging and chronic diseases. The data discussed in this article indicate that Nox2-dependent ROS may be a therapeutic target for AII-induced skeletal muscle atrophy.

## Figures and Tables

**Table 1:** Animal characteristics.

	WT + V	KO + V	WT + AII	KO + AII
*N*	*n* = 8	*n* = 6	*n* = 8	*n* = 6
Heart weight (mg)	105.0 ± 1.5	112.8 ± 2.5	138.4 ± 5.3^*∗*^	155.4 ± 3.2^*∗*^
Heart weight/body weight (mg/g)	3.8 ± 0.1	4.1 ± 0.1	5.8 ± 0.3^*∗*^	5.4 ± 0.1^*∗*^
Systolic blood pressure (mmHg)	102.0 ± 1.4	109.4 ± 2.8	149.2 ± 6.8^*∗*^	153.4 ± 5.6^*∗*^
Diastolic blood pressure (mmHg)	69.3 ± 1.7	86.7 ± 9.8	105.9 ± 4.0^*∗*^	101.4 ± 6.1^*∗*^
Mean blood pressure (mmHg)	79.1 ± 1.8	91.7 ± 2.9	120.6 ± 3.4^*∗*^	118.8 ± 5.8^*∗*^
Heart rate (bpm)	690.7 ± 13.9	687.9 ± 3.8	672.7 ± 22.9	678.0 ± 40.4

Data are expressed as means ± SEM. WT, wild type; V, vehicle; AII, angiotensin II; KO, knockout; bpm, beats per minute. ^*∗*^ *P* < 0.05 versus WT + V.

## References

[1] Cruz-Jentoft A. J., Baeyens J. P., Bauer J. M. (2010). Sarcopenia: european consensus on definition and diagnosis: report of the european working group on sarcopenia in older people. *Age and Ageing*.

[2] Braun T., Gautel M. (2011). Transcriptional mechanisms regulating skeletal muscle differentiation, growth and homeostasis. *Nature Reviews Molecular Cell Biology*.

[3] Glass D. J. (2003). Signalling pathways that mediate skeletal muscle hypertrophy and atrophy. *Nature Cell Biology*.

[4] Viña J., Salvador-Pascual A., Tarazona-Santabalbina F. J., Rodriguez-Mañas L., Gomez-Cabrera M. C. (2016). Exercise training as a drug to treat age associated frailty. *Free Radical Biology & Medicine*.

[5] Kinugawa S., Takada S., Matsushima S., Okita K., Tsutsui H. (2015). Skeletal muscle abnormalities in heart failure. *International Heart Journal*.

[6] Harada K., Sugaya T., Murakami K., Yazaki Y., Komuro I. (1999). Angiotensin II type 1A receptor knockout mice display less left ventricular remodeling and improved survival after myocardial infarction. *Circulation*.

[7] Basso N., Cini R., Pietrelli A., Ferder L., Terragno N. A., Inserra F. (2007). Protective effect of long-term angiotensin II inhibition. *American Journal of Physiology-Heart and Circulatory Physiology*.

[8] Takada S., Kinugawa S., Hirabayashi K. (2013). Angiotensin II receptor blocker improves the lowered exercise capacity and impaired mitochondrial function of the skeletal muscle in type 2 diabetic mice. *Journal of Applied Physiology*.

[9] Fukushima A., Kinugawa S., Takada S. (2016). Direct renin inhibitor ameliorates insulin resistance by improving insulin signaling and oxidative stress in the skeletal muscle from post-infarct heart failure in mice. *European Journal of Pharmacology*.

[10] Fukushima A., Kinugawa S., Takada S. (2014). (Pro)renin receptor in skeletal muscle is involved in the development of insulin resistance associated with postinfarct heart failure in mice. *American Journal of Physiology-Endocrinology and Metabolism*.

[11] Yoshida T., Delafontaine P. (2015). Mechanisms of cachexia in chronic disease states. *The American Journal of the Medical Sciences*.

[12] Kadoguchi T., Kinugawa S., Takada S. (2015). Angiotensin II can directly induce mitochondrial dysfunction, decrease oxidative fibre number and induce atrophy in mouse hindlimb skeletal muscle. *Experimental Physiology*.

[13] Griendling K. K., Minieri C. A., Ollerenshaw J. D., Alexander R. W. (1994). Angiotensin II stimulates NADH and NADPH oxidase activity in cultured vascular smooth muscle cells. *Circulation Research*.

[14] Ahn B., Beharry A. W., Frye G. S., Judge A. R., Ferreira L. F. (2015). NAD(P)H oxidase subunit p47^phox^ is elevated, and p47^phox^ knockout prevents diaphragm contractile dysfunction in heart failure. *American Journal of Physiology-Lung Cellular and Molecular Physiology*.

[15] Bechara L. R. G., Moreira J. B. N., Jannig P. R. (2014). NADPH oxidase hyperactivity induces plantaris atrophy in heart failure rats. *International Journal of Cardiology*.

[16] Cunha T. F., Bechara L. R. G., Bacurau A. V. N. (2017). Exercise training decreases NADPH oxidase activity and restores skeletal muscle mass in heart failure rats. *Journal of Applied Physiology*.

[17] Brown D. I., Griendling K. K. (2009). Nox proteins in signal transduction. *Free Radical Biology & Medicine*.

[18] Inoue N., Kinugawa S., Suga T. (2012). Angiotensin II-induced reduction in exercise capacity is associated with increased oxidative stress in skeletal muscle. *American Journal of Physiology-Heart and Circulatory Physiology*.

[19] Ono T., Takada S., Kinugawa S., Tsutsui H. (2015). Curcumin ameliorates skeletal muscle atrophy in type 1 diabetic mice by inhibiting protein ubiquitination. *Experimental Physiology*.

[20] Ohta Y., Kinugawa S., Matsushima S. (2011). Oxidative stress impairs insulin signal in skeletal muscle and causes insulin resistance in postinfarct heart failure. *American Journal of Physiology-Heart and Circulatory Physiology*.

[21] Takada S., Hirabayashi K., Kinugawa S. (2014). Pioglitazone ameliorates the lowered exercise capacity and impaired mitochondrial function of the skeletal muscle in type 2 diabetic mice. *European Journal of Pharmacology*.

[22] Takada S., Kinugawa S., Matsushima S. (2015). Sesamin prevents decline in exercise capacity and impairment of skeletal muscle mitochondrial function in mice with high-fat diet-induced diabetes. *Experimental Physiology*.

[23] Takada S., Masaki Y., Kinugawa S. (2016). Dipeptidyl peptidase-4 inhibitor improved exercise capacity and mitochondrial biogenesis in mice with heart failure via activation of glucagon-like peptide-1 receptor signalling. *Cardiovascular Research*.

[24] Suga T., Kinugawa S., Takada S. (2014). Combination of exercise training and diet restriction normalizes limited exercise capacity and impaired skeletal muscle function in diet-induced diabetic mice. *Endocrinology*.

[25] Yokota T., Kinugawa S., Hirabayashi K. (2009). Oxidative stress in skeletal muscle impairs mitochondrial respiration and limits exercise capacity in type 2 diabetic mice. *American Journal of Physiology-Heart and Circulatory Physiology*.

[26] Blendea M. C., Jacobs D., Stump C. S. (2005). Abrogation of oxidative stress improves insulin sensitivity in the Ren-2 rat model of tissue angiotensin II overexpression. *American Journal of Physiology-Endocrinology and Metabolism*.

[27] Kackstein K., Teren A., Matsumoto Y. (2013). Impact of angiotensin II on skeletal muscle metabolism and function in mice: contribution of IGF-1, Sirtuin-1 and PGC-1*α*. *Acta Histochemica*.

[28] Bodine S. C., Latres E., Baumhueter S. (2001). Identification of ubiquitin ligases required for skeletal Muscle Atrophy. *Science*.

[29] Cho H., Thorvaldsen J. L., Chu Q., Feng F., Birnbaum M. J. (2001). Akt1/PKB*α* Is Required for Normal Growth but Dispensable for Maintenance of Glucose Homeostasis in Mice. *The Journal of Biological Chemistry*.

[30] Ohanna M., Sobering A. K., Lapointe T. (2005). Atrophy of S6K1^−/−^ skeletal muscle cells reveals distinct mTOR effectors for cell cycle and size control. *Nature Cell Biology*.

[31] Aguilar V., Alliouachene S., Sotiropoulos A. (2007). S6 Kinase Deletion Suppresses Muscle Growth Adaptations to Nutrient Availability by Activating AMP Kinase. *Cell Metabolism*.

[32] Clarke B. A., Drujan D., Willis M. S. (2007). The E3 Ligase MuRF1 degrades myosin heavy chain protein in dexamethasone-treated skeletal muscle. *Cell Metabolism*.

[33] Izumiya Y., Hopkins T., Morris C. (2008). Fast/glycolytic muscle fiber growth reduces fat mass and improves metabolic parameters in obese mice. *Cell Metabolism*.

[34] Shimizu N., Yoshikawa N., Ito N. (2011). Crosstalk between glucocorticoid receptor and nutritional sensor mTOR in skeletal muscle. *Cell Metabolism*.

[35] Bendall J. K., Cave A. C., Heymes C., Gall N., Shah A. M. (2002). Pivotal role of a gp91^phox^-containing NADPH oxidase in angiotensin II-induced cardiac hypertrophy in mice. *Circulation*.

[36] Wei Y., Sowers J. R., Nistala R. (2006). Angiotensin II-induced NADPH oxidase activation impairs insulin signaling in skeletal muscle cells. *The Journal of Biological Chemistry*.

[37] Fearon K. C. H., Glass D. J., Guttridge D. C. (2012). Cancer cachexia: mediators, signaling, and metabolic pathways. *Cell Metabolism*.

[38] Trendelenburg A. U., Meyer A., Rohner D., Boyle J., Hatakeyama S., Glass D. J. (2009). Myostatin reduces Akt/TORC1/p70S6K signaling, inhibiting myoblast differentiation and myotube size. *American Journal of Physiology-Cell Physiology*.

[39] Wang B.-W., Chang H., Kuan P., Shyu K.-G. (2008). Angiotensin II activates myostatin expression in cultured rat neonatal cardiomyocytes via p38 MAP kinase and myocyte enhance factor 2 pathway. *Journal of Endocrinology*.

[40] Gómez-Mauricio G., Moscoso I., Martín-Cancho M.-F. (2016). Combined administration of mesenchymal stem cells overexpressing IGF-1 and HGF enhances neovascularization but moderately improves cardiac regeneration in a porcine model. *Stem Cell Research & Therapy*.

[41] Russell S. T., Eley H., Tisdale M. J. (2007). Role of reactive oxygen species in protein degradation in murine myotubes induced by proteolysis-inducing factor and angiotensin II. *Cellular Signalling*.

[42] Semprun-Prieto L. C., Sukhanov S., Yoshida T. (2011). Angiotensin II induced catabolic effect and muscle atrophy are redox dependent. *Biochemical and Biophysical Research Communications*.

[43] Li Y.-P., Chen Y., Li A. S., Reid M. B. (2003). Hydrogen peroxide stimulates ubiquitin-conjugating activity and expression of genes for specific E2 and E3 proteins in skeletal muscle myotubes. *American Journal of Physiology-Cell Physiology*.

[44] Haegens A., Schols A. M., Gorissen S. H. (2012). NF-_*κ*B activation and polyubiquitin conjugation are required for pulmonary inflammation-induced diaphragm atrophy. *American Journal of Physiology-Lung Cellular and Molecular Physiology*.

[45] Li Y., Chen Y., John J. (2005). TNF-*α* acts via p38 MAPK to stimulate expression of the ubiquitin ligase atrogin1/MAFbx in skeletal muscle. *The FASEB Journal*.

[46] Mittal A., Bhatnagar S., Kumar A. (2010). The TWEAK-Fn14 system is a critical regulator of denervation-induced skeletal muscle atrophy in mice. *The Journal of Cell Biology*.

[47] Li P., Li Z. (2015). Neuroprotective effect of paeoniflorin on H2O2-induced apoptosis in PC12 cells by modulation of reactive oxygen species and the inflammatory response. *Experimental and Therapeutic Medicine*.

[48] Pollock J. D., Williams D. A., Gifford M. A. C. (1995). Mouse model of X-linked chronic granulomatous disease, an inherited defect in phagocyte superoxide production. *Nature Genetics*.

[49] Kinugawa S., Zhang J., Messina E. (2005). Gp91phox-containing NAD(P)H oxidase mediates attenuation of nitric oxide-dependent control of myocardial oxygen consumption by ANG II. *American Journal of Physiology-Heart and Circulatory Physiology*.

